# Supplementary material for: Haplotype threading: accurate polyploid phasing from long reads
Source: Genome Biol. 2020 Sep 21;21:252. doi: 10.1186/s13059-020-02158-1 (PMC7504856; doi:10.1186/s13059-020-02158-1)
Supplement: Supplementary file 1 — Additional file 1 We provide the switch errors and Hamming rates of WhatsHap polyphase and H-PoPG, HapCompass, HapTree and Ranbow on an exemplary region of chromosome 1. Further, we provide figures showing the N50 block lengths and the corresponding Hamming rates for different block cut strategies of WhatsHap polyphase on the artificial tetraploid human dataset, including real and simulated reads on different coverages. We also include a comparison between WhatsHap polyphase and H-PoPG in collapsing regions, using long blocks similar to H-PoPG. For the potato data, we show the relation between the fraction of phased variants and heterozygosity level in the potato genes and also include a figure showing the haplotype assemblies for the FRIGIDA-like protein 5 isoform X2 gene. Additionally, we show an example for the phasing behavior of WhatsHap polyphase and H-PoPG over weakly connected variants. Last, we provide a description of the haploid N50 block length and the comparison between the regular and the haploid N50 for several simulated datasets. [file 13059_2020_2158_MOESM1_ESM.pdf]

Haplotype Threading: accurate polyploid phasing from long reads - Supplementary Material

Sven D. Schrinner, Rebecca Serra Mari, Jana Ebler, Mikko Rautiainen, Lancelot Seillier, Julia J. Reimer, Björn Usadel, Tobias Marschall, Gunnar W. Klau

1 Comparison between WhatsHap polyphase and several competitors on an exemplary region of Chromosome 1

Table S1: Comparison between the resulting switch error rates of WhatsHap polyphase (using the configuration for longer blocks), H-PoPG, HapCompass, HapTree and Ranbow on a 1 Mb region (40Mb-41Mb) of Chromosome 1 from our simulated and real tetraploid datasets (coverage 40×).

method	SER (%)	HR (%)
WH-PP*	1.36	22.08
H-PoPG	1.52	17.06
Ranbow	error	
HapCompass	12.31	31.48
HapTree	error	

(a) real read data

method	SER (%)	HR (%)
WH-PP*	0.38	18.83
H-PoPG	1.79	20.63
Ranbow	17.87	25.44
HapCompass	13.06	32.01
HapTree	error	

(b) simulated read data

2 Hamming rates for artificial tetraploid human

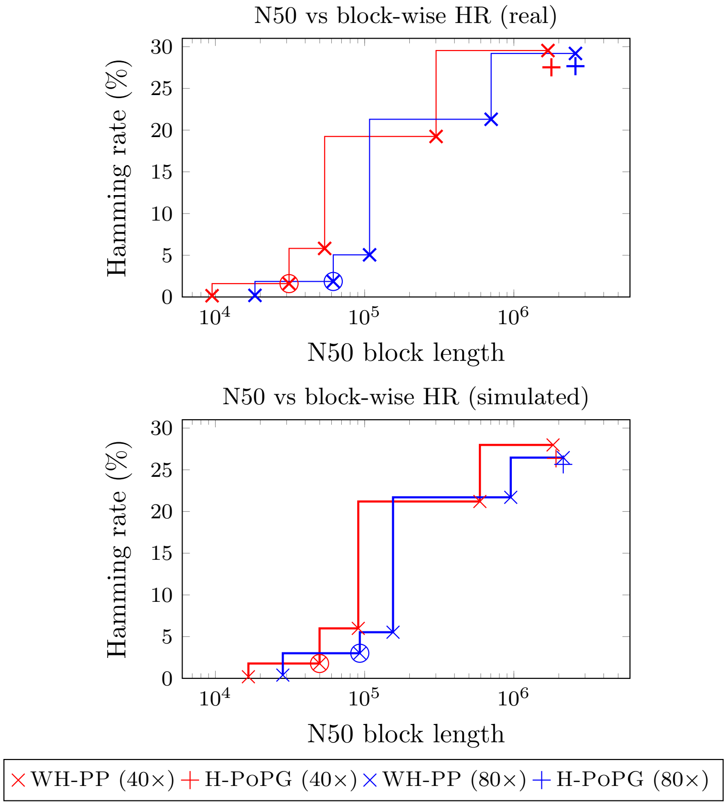

Figure S1: N50 block lengths and the respective block-wise Hamming rates for different block cut strategies of WhatsHap polyphase (default strategy marked by a circle) on the tetraploid real read dataset (top) and the simulated dataset (bottom) with 40× and 80× coverage. Note that Hamming rates above 10 or 20 percent do not seem to be useful in practice. If one used such a phasing to query whether two alleles lie on the same haplotype, the chance of error would be as high. The results illustrate that both methods are unable to produce good phasings over longs blocks. WhatsHap polyphase achieves reasonably low Hamming error rates using the default block cut strategy.

3 Comparison between H-PoPG and WhatsHap polyphase in collapsing regions, using long blocks similar to H-PoPG

Table S2: Comparison between the resulting switch error rates of H-PoPG and WhatsHap polyphase using block lengths that are comparable to H-PoPG (WH-PP*) on collapsing regions over at least 50 variants as compared to non-collapsing regions and the average throughout the genome. Results (switch error rates in %) are presented for Chromsome 1 of the real (a) and the simulated (b) dataset, testing 40× and 80× coverage. The third row marks the quotient between the switch error rate of H-PoPG and that of WhatsHap polyphase to highlight by which magnitude the results differ.

coverage	method	collapsing regions	non-collapsing regions	total
40×	WH-PP*	0.66	1.81	1.65
	H-PoPG	2.02	2.16	2.02
	SER($\frac{\text{H-PoPG}}{\text{WH-PP}}$)	3.06	1.19	1.22
80×	WH-PP*	0.38	1.16	0.99
	H-PoPG	1.05	1.30	1.24
	SER($\frac{\text{H-PoPG}}{\text{WH-PP}}$)	2.76	1.12	1.25

(a) real read data

coverage	method	collapsing regions	non-collapsing regions	total
40×	WH-PP*	0.45	1.29	1.19
	H-PoPG	2.01	1.63	1.68
	SER($\frac{\text{H-PoPG}}{\text{WH-PP}}$)	4.47	1.62	1.41
80×	WH-PP*	0.25	0.88	0.82
	H-PoPG	0.94	0.98	0.99
	SER($\frac{\text{H-PoPG}}{\text{WH-PP}}$)	3.76	1.11	1.21

(b) simulated read data

4 Phased variants in largest block in relation to heterozygosity level

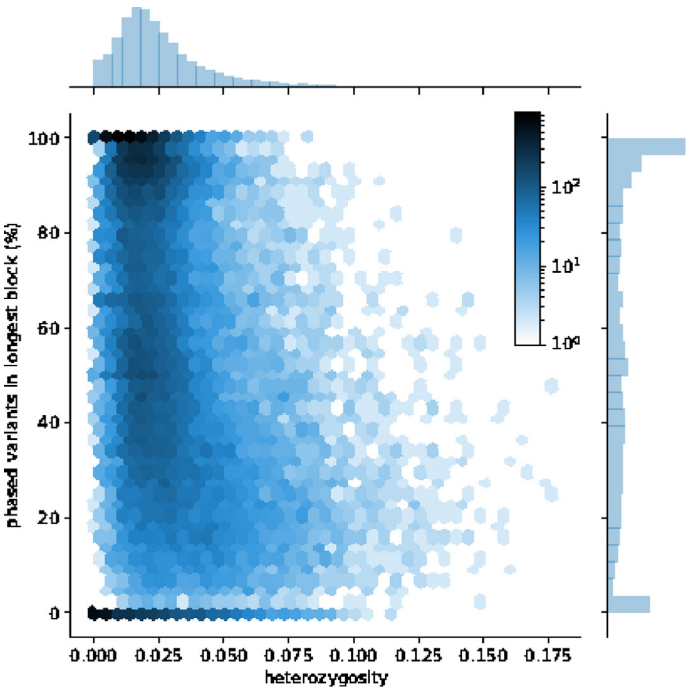

Figure S2: **Fraction of phased variants in relation to heterozygosity.** We determine the heterozygosity level of a gene as the fraction of heterozygous positions. The plot shows the fraction of phased variants (y-axis) in relation to heterozygosity level (x-axis). Axis histograms and hexagons illustrate the distribution of data points.

5 Alignment of the haplotype sequences of the FRIGIDA gene and the reference genome

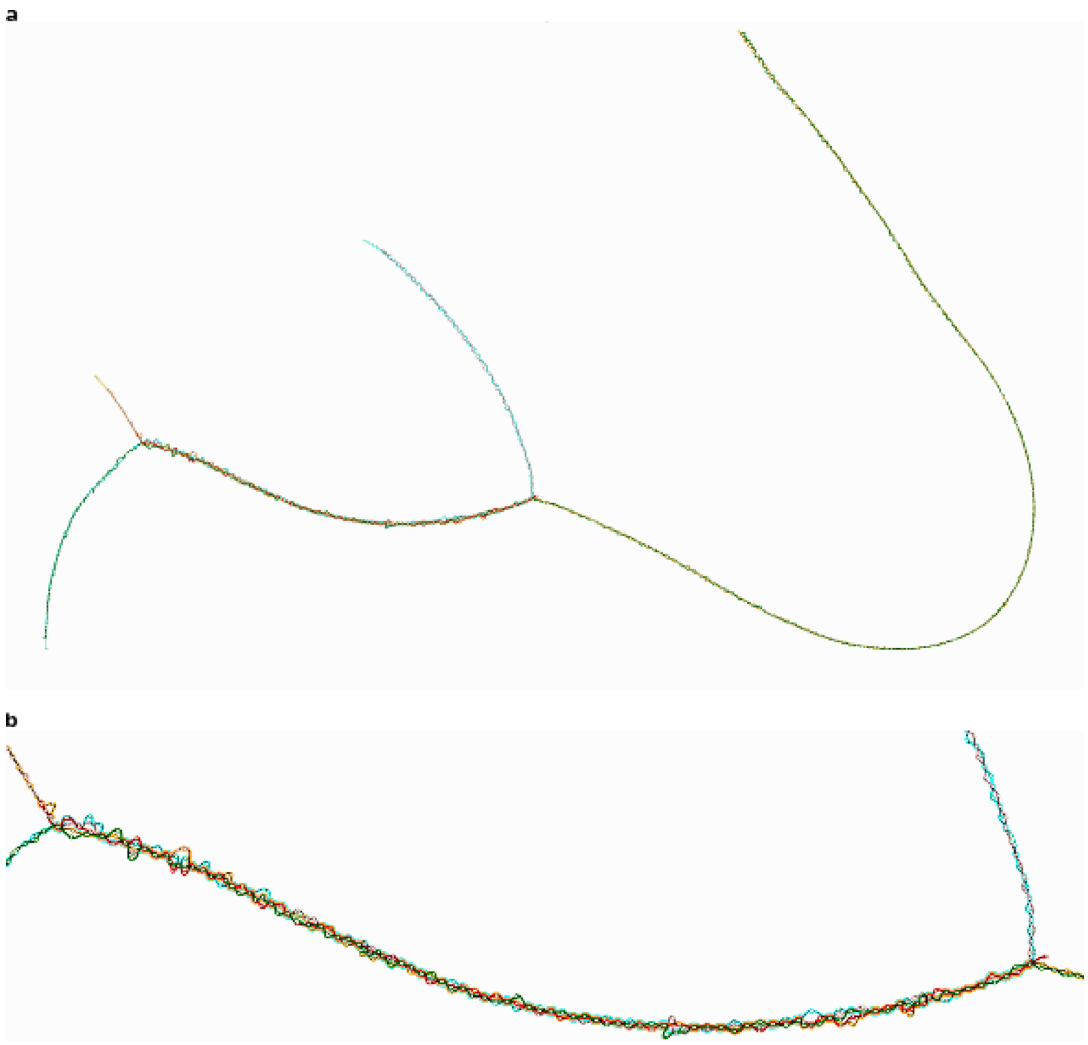

Figure S3: **Haplotype assemblies for the FRIGIDA gene.** We ran Reveal (`https://github.com/jasperlinthorst/reveal`) to produce a graph that represents an alignment of the local haplotype assemblies for the FRIGIDA gene and the corresponding reference sequence. We visualized this graph using GfaViz [1]. The red sequence corresponds to the reference genome. **a)** shows the whole graph, **b)** shows the part of the graph that corresponds to the FRIGIDA-like protein 5 isoform X2 gene.

6 Phasing blocks over weakly connected variants

The phasings from H-PoPG and the alternative configuration of WhatsHap polyphase connect variants that are only linked by a single read as shown in Figure S4. The variants at positions 13744460 and 13763903 (left and right border of the shown region) are so far apart that only one read covers both, yet H-PoPG phases both variants in a single block. While this can work in the diploid case, where one haplotype is the complement of the other (assuming heterozygous sites only), the polyploid case requires more connectivity for confident phasings.

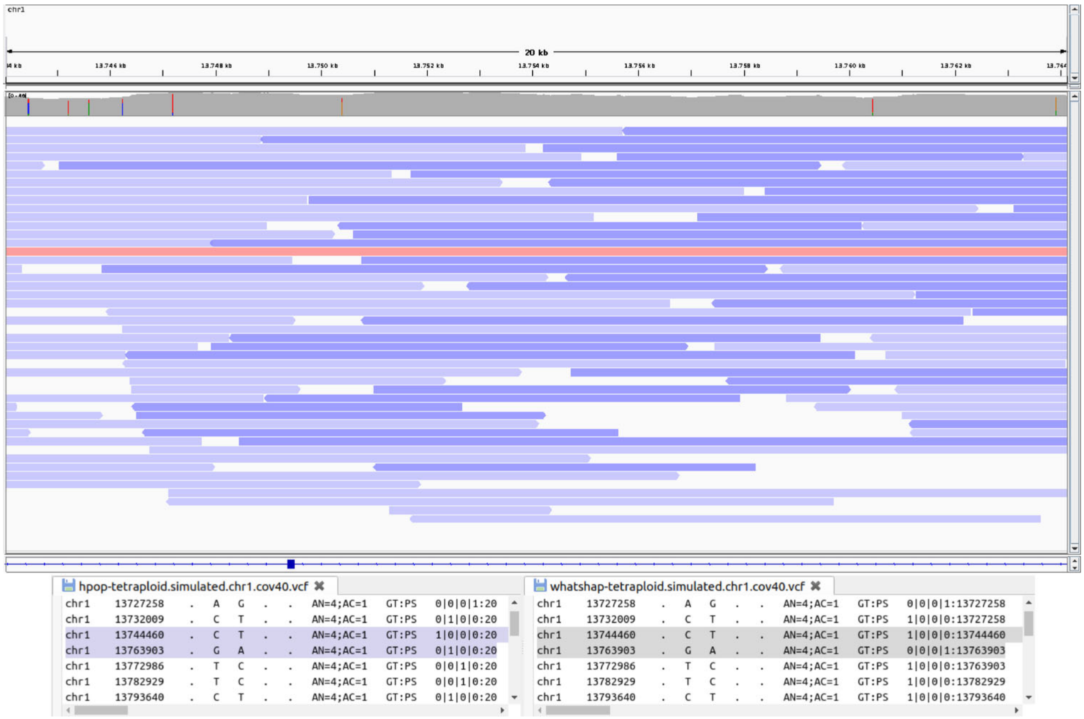

Figure S4: **Read alignments between phasing blocks.** The screenshot from IGV shows the read alignments between two variants located at positions 13744460 and 13763903. Only one read (colored red) covers both variants.

7 Haploid phasing blocks

Motivated by the fact that not all haplotypes need to be cut every time, when there is ambiguity between a subset of haplotypes at a certain location, one can look at the *haploid N50 block length.* This is a generalization of the regular N50 block length, where the blocks from each haplotype are taken individually and where the blocks not only have to cover 50% of the chromosome length, but 50% of chromosome length times the number haplotypes. If all haplotypes are cut at the same positions, this is equivalent to the regular N50 block length, but it can be higher if the blocks overlap.

We calculated the haploid N50 block length for the simulated datasets in Table S3 to show that the blocks in each haplotype are longer than the regular N50 implies. The effect becomes stronger for higher ploidies. However, we note here that these larger N50 values are not to be mixed with the results from Table 1, because the error rates refer to the old block partition of the phasing. Although the defined error rates cannot be trivially generalized for overlapping blocks, we expect more errors in the phasing simply because the individual haplotype blocks are longer.

Table S3: Comparison between regular and haploid N50 block lengths for the simulated datasets.

dataset	N50	haploid N50
tetraploid 40X	48815	86973
tetraploid 80X	86227	156041
pentaploid 40X	22625	44365
pentaploid 80X	33438	68263
hexaploid 40X	16785	37609
hexaploid 80X	26711	65621

References

[1] Giorgio Gonnella, Niklas Niehus, Stefan Kurtz. GfaViz: flexible and interactive visualization of GFA sequence graphs, *Bioinformatics*, 35(16):2853–2855, 2019.
