# Supplementary material for: Haplotype threading: accurate polyploid phasing from long reads
Source: Genome Biol. 2020 Sep 21;21:252. doi: 10.1186/s13059-020-02158-1 (PMC7504856; doi:10.1186/s13059-020-02158-1)
Supplement: Supplementary file 2 — Additional file 2 Review history. [file 13059_2020_2158_MOESM2_ESM.docx]

Review History

**First round of review**

**Reviewer 1**

**Were you able to assess all statistics in the manuscript, including the appropriateness of statistical tests used?**

Yes: not many statistical results are shown.

**Were you able to directly test the methods?**

No

**Comments to author:**

The authors of the manuscript "Haplotype Threading: Accurate Polyploid Phasing from Long Reads" describe an extension of Whatshap that allows to phase polyploid genomes. This is mainly based on a new clustering approach (MEC) that achieves better performances in regions where a subset of the haplotypes might be similar to identical. They benchmark their new method based on simulated /synthetic data set for the human chromosome 1 and over a real data set from potato. This is carried out mainly based on the switch error rate and the hemming distance with respect to the truth set. Overall the manuscript is well written and easy to follow. In the following I list my questions and concerns.

1.     I appreciate the detailed introduction and explanation of the available methods. However, I am wondering if this is a bit too long and the space might be better used by exploring the performance of Whatshapp polyploid over different long read data / HiC. This would give the reader valuable insights what would work better for his or her organism.

2.     I would encourage you to also include another example of a tetra ploid or higher ploidy. One example data set that could work for this is the grape vine used in the Falcon Unzip paper(https://doi.org/10.1038/nmeth.4035 )

3.     Maybe also include other programs as you are mentioning them already. I am not asking for benchmarking all of them but maybe 2-3 other programs would give a more comprehensive insight in the performance improvement of your method.

4.     I am wondering how the concept of phase block works on higher polyploidy. Maybe this is a naïve question, but is a phase block still the region of confident phasing for all the haplotypes or will there be changes if 2 out of the 4 haplotypes can be easily distinguished ?

5.     It would be interesting to further see the length of the genes that you phase  (Figure 4b) and or their heterozygosity level.

6.     You mention multiple times that Whatshap polyphase can be adjusted for larger N50. However, you do not show concrete numbers of the performance compared to H-PopG. It would be great to see the performance of Whatshap polyphase for a similar N50 in Table 1 and 2

7.     I would further encourage you to simulate higher ploidy genomes and uneven numbers as well. This program aims at solving an important problem for polyploid genomes, but so far you only demonstrate it on tetraploid samples.

8.     Maybe you can elaborate why you think the switch errors are higher in simulated data vs. real data. Is this driven by the organisms (chr1 from human vs. potato? ) or some read lengths etc?

9.     I am further wondering if Whatshap polyphase would be able to phase over regions of different polyploidy within the same genome?

10.     Figure 3: The symbols are hard to read. Maybe if you enlarge them that would help. This is especially the case for these under the lines.

**Reviewer 2**

**Were you able to assess all statistics in the manuscript, including the appropriateness of statistical tests used?**

Yes: The appropriate statistical tests were used

**Were you able to directly test the methods?**

No

**Comments to author:**

Schrinner et al. present a new algorithm, WhatsHap polyphase (WH-PP), for phasing haplotypes in polyploids using long read data. WH-PP is advantageous over other haplotype phasing programs as it first clusters reads using position-dependent scoring and then stitches the haplotypes across clusters using coverage with dynamic programing. WH-PP stringently cuts haplotype blocks at junctions with low coverage or few distinguishing polymorphisms, resulting in higher overall accuracy. The authors tested WH-PP using simulated and real data from human chromosome 1 and Nanopore data for tetraploid potato. Overall, WH-PP performed better than the current standard J-PoPG, especially in collapsed regions with low heterozygosity. WH-PP is implemented in the widely used WhatsHap, making it easily accessible by the community. This approach seems technically sound, but I have a few concerns about the performance and utility of this approach that should be addressed.

1. The main argument for using WH-PP over other programs is the low switch error rate, but the massive differences in default block N50s make this claim slightly misleading. The haplotype blocks produced by WH-PP have higher accuracy than J-PoPG, but a ~fifty-fold lower block N50 for only a 61-68% reduction in stitch error rate. The sensitivity of WH-PP is advantageous but the utility is low with such short haplotype blocks. The authors state the error rates are comparable when block N50s are similar, but in Supplemental Figure 7, it seems WH-PP performs worse than H-PoPG on real data with comparable block lengths. WH-PP is also considerably more computationally intensive than H-PoPG. This scales exponentially with ploidy increases, making it challenging to phase genomes with more than 6x ploidy. Overall, I think the manuscript could be improved if the strengths and limitations of WH-PP were highlighted better.

2. Broadly, it is unclear how well WH-PP performed on the potato data. Plants are generally more difficult to phase than animals given their uneven rates of within genome heterozygosity, structural variants, TEs, etc. Only 11 % of potato genes were completely phased into a single block and most genes were partially phased. This is a massive computational challenge, but all four phased haplotypes could likely be assembled separately using the same raw Nanopore data with a phase aware assembler in much larger blocks. Data on the FRIGIDA gene shows haplotypes are nicely phased, but this is a single gene spanning only a few kb. Global metrics and more detailed comparisons would strengthen the manuscript and encourage readers to use this approach over others. Pre-filtering, error correction, alignment, and variant calling of Nanopore data as outlined is quite intensive, so a strong justification should be made.

It would be useful to report the statistics for tetraploid potato haplotype phasing for WH-PP and H-PoPG in a summary table similar to table 1. It's difficult to assess how well WH-PP performed without additional metrics.

3. For testing WH-PP in potato, the Nanopore reads were first error corrected using Illumina data prior to haplotype phasing. This approach can be problematic as it is difficult to unambiguously align short Illumina reads to the raw Nanopore reads, and this can lead to over polishing, removal of true variation or creating chimeric haplotypes in individual reads. This does not seem to be a problem based on the visualization in Figure 4, but some metric would be helpful.

Minor:

Figure 1: Should the figure legend read "larger stretches of homozygous sites" instead of ""larger stretches of heterozygous sites"? Haplotypes with long runs of homozygosity would lead to phasing issues, as no variants would distinguish them. Perhaps I misinterpreted the wording.

**Authors Response**

**Point-by-point responses to the reviewers’ comments:**

Reviewer 1

The authors of the manuscript "Haplotype Threading: Accurate Polyploid Phasing from Long Reads" describe an extension of Whatshap that allows to phase polyploid genomes. This is mainly based on a new clustering approach (MEC) that achieves better perfor-mances in regions where a subset of the haplotypes might be similar to identical. They benchmark their new method based on simulated /synthetic data set for the human chro-mosome 1 and over a real data set from potato. This is carried out mainly based on the switch error rate and the hemming distance with respect to the truth set. Overall the man-uscript is well written and easy to follow. In the following I list my questions and concerns.

1. I appreciate the detailed introduction and explanation of the available methods. However, I am wondering if this is a bit too long and the space might be better used by exploring the performance of Whatshapp polyploid over different long read data / HiC. This would give the reader valuable insights what would work better for his or her organism.

*We slightly shortened the introduction and additionally ran experiments on long read data sets with higher ploidy.*

2. I would encourage you to also include another example of a tetra ploid or higher ploidy. One example data set that could work for this is the grape vine used in the Falcon Unzip paper (https://doi.org/10.1038/nmeth.4035)

*We thank the reviewer for the suggestion to include higher ploidies in our study. We reached out to Jason Chin (the author of Falcon-Unzip) and according to him, the grape cultivar sequenced as part of the Falcon-Unzip paper is diploid, and there were no polyploids included in that study. To still be able to test WhatsHap polyphase on polyploid genomes, we constructed penta- and hexaploid sequencing data sets by performing in silico mixing of real human sequencing data. The results are now included in Table 1. We now show that our tool is able to run on higher ploidies in reasonable time and that the error rates remain better than those of H-PoPG.*

3. Maybe also include other programs as you are mentioning them already. I am not asking for benchmarking all of them but maybe 2-3 other programs would give a more comprehensive insight in the performance improvement of your method.

*In our initial submission we focused on the comparison with H-PoPG, because other programs were difficult or impossible to run on our data, even with significant effort, and H-PoPG yielded promising results in preliminary tests. We now tried three additional tools (HapCompass, HapTree and Ranbow) as well as the new version of H-Pop on our data.*

*HapCompass: On our datasets, HapCompass takes too much time. We stopped the computations of HapCompass after four days on the 40x Chr1 tetraploid dataset.*

*HapTree does not output haplotype blocks but has to be run on connected blocks, which we had to precompute. Nevertheless, HapTree crashed on this data. As the source code is not available, we could not debug the error. We contacted the authors in June but did not hear back so far.*

*We also had to adapt the Ranbow code to run on our data. Since Ranbow focuses on short read data it ran, as expected, into trouble and took excessive time on the long read data.*

*In the meantime, a new version of H-PoPG was released, and we replaced the results in the paper by those obtained with the new version. The stats, however, changed only marginally.*

*Because of the problems of the additional tools with the full data we focused on a 1Mb region of Chr1, which we included as Suppl. Table 3. Again, we observed some crashes, and, where not, the tools resulted in low quality phasings with switch error rates strongly above 10%.*

4. I am wondering how the concept of phase block works on higher polyploidy. Maybe this is a naïve question, but is a phase block still the region of confident phasing for all the haplotypes or will there be changes if 2 out of the 4 haplotypes can be easily distinguished?

*This is an excellent question. Presently the standard encoding of phased blocks in the VCF format prescribes common block boundaries for all haplotypes. However, the haplotype threading procedure we introduce in this paper can in fact distinguish which haplotypes have been reconstructed with confidence and which haplotypes are uncertain, which manifests as a switch from one cluster to another. Even though not standard VCF, we have now augmented the output to also record which haplotypes switch for each of the block boundaries. This leads to longer phasing blocks (~1.8 to 2.4 times as long) inside individual haplotypes, but this concept requires new quality metrics to compare it to the error rates of the standard phasing blocks.*

5. It would be interesting to further see the length of the genes that you phase (Figure 4b) and or their heterozygosity level.

*We changed Figure 4b such that it now also shows the length of the phased genes and added another plot (Suppl. Fig. 8) that shows the fraction of phased variants in relation to the heterozygosity level of the genes. The plots show that we can fully phase a large number of genes, including long ones, and that the level of heterozygosity does not have a strong effect on the percentage of variants we can phase in the longest block.*

6. You mention multiple times that Whatshap polyphase can be adjusted for larger N50. However, you do not show concrete numbers of the performance compared to H-PopG. It would be great to see the performance of Whatshap polyphase for a similar N50 in Table 1 and 2

*The different block cut configuration for WhatsHap are indicated in Figure 3, where each of the x-symbols resembles one such configuration. The x-marks with circles around them are the data previously shown in Table 1. We now extended Table 1 with the configuration that is closest to HPoPG in terms of N50. In both tools (HPoPG and the corresponding configuration of WhatsHap) this results in phasing blocks only being cut when there is no read at all connecting two heterozygous variants (with slight deviations because of different internal alignment handling). Despite the good N50 values, this policy has severe drawbacks as we now illustrate by means of an IGV screenshot in Suppl. Fig. 10. If only one read connects two variants, it is actually impossible to properly phase more than two haplotypes.*

7. I would further encourage you to simulate higher ploidy genomes and uneven numbers as well. This program aims at solving an important problem for polyploid genomes, but so far you only demonstrate it on tetraploid samples.

*In addition to the simulated tetraploid data, we mixed in a third human sample (HG00733) to create pentaploid and hexaploid data as well, each with both 40X and 80X coverage. We ran our experiments on the new data and added the results to Table 1. As expected, the error rates increase for all tools. In fact, the improvement of WhatsHap over H-PoPG with respect to switch errors becomes even larger with higher ploidy. It should be noted, however, that the N50s for WhatsHap become smaller compared to the tetraploid samples. This is related to the issue discussed in the reply to comment 4: The more haplotypes there are, the higher the chance that a cut is made between two variants by the stringent block cut strategy we recommend as a default setting, resulting in shorter blocks. On the other hand, the issue of H-PoPG (overphasing), described in the reply to comment 6, also becomes more severe with increasing ploidy.*

8. Maybe you can elaborate why you think the switch errors are higher in simulated data vs. real data. Is this driven by the organisms (chr1 from human vs. potato? ) or some read lengths etc?

*We have computed switch errors only for our synthetic human datasets where a ground truth is available. Here, we actually observe lower errors for simulated data which is prob-ably due to the read simulators which seem to create slightly easier to phase reads.*

9. I am further wondering if Whatshap polyphase would be able to phase over regions of different polyploidy within the same genome?

*This is a great point and we had discussed such a functionality earlier internally. To do that, one would presently have to determine such regions of different ploidy externally, e.g. by running a coverage-based copy number caller, and then run WhatsHap separately on each regions. We aim to integrate such a copy number calling mechanism directly into WhatsHap in the future and now mention this idea in the Conclusions section:*

*"Our haplotype threading framework is well suited to be extended to use local ploidy esti-mates. That would allow phasing genomic regions where the observed ploidy deviates, for instance due to aneuploidies, large deletions on a subset of haplotypes or collapses of segmental duplications in the used reference genome, and we plan to add this functionality to future releases."*

10. Figure 3: The symbols are hard to read. Maybe if you enlarge them that would help. This is especially the case for these under the lines.

*We made the symbols in the plots larger and also the pareto lines a bit thinner. We also changed the diamonds into plus symbols to show the actual data point more precisely.*

Reviewer 2

Schrinner et al. present a new algorithm, WhatsHap polyphase (WH-PP), for phasing hap-lotypes in polyploids using long read data. WH-PP is advantageous over other haplotype phasing programs as it first clusters reads using position-dependent scoring and then stitches the haplotypes across clusters using coverage with dynamic programing. WH-PP stringently cuts haplotype blocks at junctions with low coverage or few distinguishing polymorphisms, resulting in higher overall accuracy. The authors tested WH-PP using simulated and real data from human chromosome 1 and Nanopore data for tetraploid potato. Overall, WH-PP performed better than the current standard J-PoPG, especially in col-lapsed regions with low heterozygosity. WH-PP is implemented in the widely used WhatsHap, making it easily accessible by the community. This approach seems technically sound, but I have a few concerns about the performance and utility of this approach that should be addressed.

1. The main argument for using WH-PP over other programs is the low switch error rate, but the massive differences in default block N50s make this claim slightly misleading. The haplotype blocks produced by WH-PP have higher accuracy than J-PoPG, but a ~fifty-fold lower block N50 for only a 61-68% reduction in stitch error rate. The sensitivity of WH-PP is advantageous but the utility is low with such short haplotype blocks. The authors state the error rates are comparable when block N50s are similar, but in Supplemental Figure 7, it seems WH-PP performs worse than H-PoPG on real data with comparable block lengths. WH-PP is also considerably more computationally intensive than H-PoPG. This scales exponentially with ploidy increases, making it challenging to phase genomes with more than 6x ploidy. Overall, I think the manuscript could be improved if the strengths and limitations of WH-PP were highlighted better.

*We extended our experiments with simulated pentaploid and hexaploid data sets and also reran the old experiments, because WhatsHap got several updates since the first submis-sion. The pentaploid and hexaploid data was created by extending the previous artificial tetraploid sample with a third human sample (HG00733) for which we have a gold standard phasing.*

*Concerning the running time: It is true that HPoPG needs less time on the original tetraploid samples and this did not change. For higher ploidy the running time scales exponentially in the worst case, but in our experiments the running time compared to HPoPG was overall not worse than on the tetraploid data. We added the higher running time to our discussion. In addition we also added the memory consumption, which ended up being lower that HPoPG.*

*Concerning the block lengths: We added a second configuration of WhatsHap to Table 1 in order to make the N50s more comparable between WhatsHap and HPoPG. In terms of switch error rate, WhatsHap remains better than HPoPG, while the Hamming rate is overall slightly worse. The high Hamming rate, however, is the reason why we believe that the long-block-phasings are not good. HPoPG only cuts the phasing when two variants are not connected by any read. For a polyploid phasing this is problematic as the information of a single read is not sufficient anymore, compared to diploid phasing. We added a figure to the supplement (Suppl. Fig. 10) that illustrates this problem.*

2. Broadly, it is unclear how well WH-PP performed on the potato data. Plants are generally more difficult to phase than animals given their uneven rates of within genome heterozygosity, structural variants, TEs, etc. Only 11 % of potato genes were completely phased into a single block and most genes were partially phased. This is a massive computational challenge, but all four phased haplotypes could likely be assembled separately using the same raw Nanopore data with a phase aware assembler in much larger blocks. Data on the FRIGIDA gene shows haplotypes are nicely phased, but this is a single gene spanning only a few kb. Global metrics and more detailed comparisons would strengthen the manuscript and encourage readers to use this approach over others. Pre-filtering, error correc-tion, alignment, and variant calling of Nanopore data as outlined is quite intensive, so a strong justification should be made.

It would be useful to report the statistics for tetraploid potato haplotype phasing for WH-PP and H-PoPG in a summary table similar to table 1. It's difficult to assess how well WH-PP performed without additional metrics.

*This is a valid concern and indeed it had been our hope that haplotype resolution would be possible through assembly. Indeed when assembling the raw data we get genome assemblies that are larger than the haploid genome size. But in no case however, we came close to even 2x the haploid genome size. Hence phasing is at best spurios and falls short of the expected 3-4x the haploid genome size. One reason for this is of course that Oxford Nanopore data is highly error prone and contains uncorrectable errors that makes phase-aware assembly more difficult. In particular, even in highly heterozygous species like potato the error rate is much higher than the heterozygosity rate. That implies that read overlap criteria used by genome assembly tools will produce links between reads from different haplotypes, leading to the observed collapse of different haplotypes.*

*As a possible way to circumvent this problem, we also tried genome assembly from the Illumina-corrected ONT reads. To this end, we tested assembly tools (e.g. Hifiasm, https://github.com/chhylp123/hifiasm) designed for highly accurate long reads (PacBio Hifi), which use stringent overlap criteria suitable for such accurate read sets. For this ex-periment, we removed any corrected reads smaller than 10kbases and separately any corrected reads smaller than 2kbases. In both cases, however, we were not able to get an assembly larger than 200 Mbases thus representing ~1/4 of the haploid potato genome size only.*

*In summary, we find that, while haplotype-specific de novo assembly is an attractive option in general, there is presently no tool/workflow applicable to our data in a polyploid setting. For a diploid setting we have recently introduced a corresponding workflow (Porubsky, Ebert, et al., Nature Biotechnology, in press; preprint: https://doi.org/10.1101/855049), that partitions reads by phasing them with respect to a haplotype-collapsed ("squashed") assembly. Notably, this requires phasing with respect to a "reference", which in this case is given by the squashed assembly. Hence extending such workflows to the polyploid case will require reference-based polyploid phasing as a sub-task.*

*Unfortunately, we lack a ground truth data set for potato and hence are unable to provide an evaluation/comparison of phasing performance similar to Table 1.*

3. For testing WH-PP in potato, the Nanopore reads were first error corrected using Illumina data prior to haplotype phasing. This approach can be problematic as it is difficult to unambiguously align short Illumina reads to the raw Nanopore reads, and this can lead to over polishing, removal of true variation or creating chimeric haplotypes in individual reads. This does not seem to be a problem based on the visualization in Figure 4, but some metric would be helpful.

*We would like to emphasize that the used error correction procedure does not align Illumina reads to the ONT reads, but instead aligns ONT reads into de Bruijn graphs built from the Illumina reads. That is a big difference and avoids the problem highlighted by the re-viewer. While this principle was introduced already in 2014 by Salmela and Rivals (doi:10.1093/bioinformatics/btu538), the much faster runtimes and better alignment accuracy of GraphAligner render this approach usable in practice. Given that a detailed evaluation of the error correction accuracy is provided in the cited preprint (https://www.biorxiv.org/content/10.1101/810812v1), we believe that repeating similar experiments here would be out of scope of the present paper.*

Minor:

Figure 1: Should the figure legend read "larger stretches of homozygous sites" instead of ""larger stretches of heterozygous sites"? Haplotypes with long runs of homozygosity would lead to phasing issues, as no variants would distinguish them. Perhaps I misinterpreted the wording.

*Thanks for the comment. It is true that long stretches of homozygous sites cause trouble for all phasing algorithms, which only use the information of heterozygous sites. Here, we consider the case of larger stretches of "locally homozygous sites", shared by two or more haplotypes. We removed the word "heterozygous" from the figure caption in order to make this more clear.*

*Yours sincerely,*

*on behalf of my co-authors*

*Gunnar W. Klau, Heinrich Heine University Düsseldorf*

**Second round of review**

**Reviewer 1**

The authors have answered all my questions and concerns and incorporated them into the main text.

I think by that it helped improve the manuscript further. I dont have any more questions or concerns.

Thank you

Fritz Sedlazeck

**Reviewer 2**

The authors have addressed my previous concerns in their revised manuscript.
